# A possible cranio-oro-facial phenotype in Cockayne syndrome

**DOI:** 10.1186/1750-1172-8-9

**Published:** 2013-01-14

**Authors:** Agnès Bloch-Zupan, Morgan Rousseaux, Virginie Laugel, Matthieu Schmittbuhl, Rémy Mathis, Emmanuelle Desforges, Mériam Koob, Ariane Zaloszyc, Hélène Dollfus, Vincent Laugel

**Affiliations:** 1 Faculté de Chirurgie Dentaire de Strasbourg, Université de Strasbourg, 1 place de l’Hôpital, Strasbourg, 67000, France; 2 Reference Centre for Oro-dental Manifestations of Rare Diseases, Pôle de Médecine et Chirurgie Bucco-Dentaires, Hôpitaux Universitaires de Strasbourg (HUS), Strasbourg, 67000, France; 3 IGBMC (Institute of Genetics and Molecular and Cellular Biology), INSERM, U964; CNRS, UMR7104, Illkirch, 67400, France; 4 Eastman Dental Institute, University College London, London, UK; 5 INSERM UMR 977 "Biomaterials and Tissue Engineering", Faculty of Dentistry, University of Strasbourg, Strasbourg, France; 6 Department of Radiology, HUS, Strasbourg, France; 7 Service de Génétique Médicale, Hôpitaux Universitaires de Strasbourg, Strasbourg, 67000, France; 8 Laboratoire Physiopathologie des syndromes rares héréditaires, Equipe EA 3949 INSERM-AVENIR, Université de Strasbourg, Faculté de Médecine, 11 rue Humann, Strasbourg, 67000, France

**Keywords:** Cockayne Syndrome, Phenotype, Tooth development, Tooth abnormalities, Cephalometry, ERCC6, ERCC8

## Abstract

**Background:**

Cockayne Syndrome CS (Type A – CSA; or CS Type I OMIM #216400) (Type B – CSB; or CS Type II OMIM #133540) is a rare autosomal recessive neurological disease caused by defects in DNA repair characterized by progressive cachectic dwarfism, progressive intellectual disability with cerebral leukodystrophy, microcephaly, progressive pigmentary retinopathy, sensorineural deafness photosensitivity and possibly orofacial and dental anomalies.

**Methods:**

We studied the cranio-oro-facial status of a group of 17 CS patients from 15 families participating in the National Hospital Program for Clinical Research (PHRC) 2005 « Clinical and molecular study of Cockayne syndrome ». All patients were examined by two investigators using the Diagnosing Dental Defects Database (D[4]/phenodent) record form.

**Results:**

Various oro-facial and dental anomalies were found: retrognathia; micrognathia; high- arched narrow palate; tooth crowding; hypodontia (missing permanent lateral incisor, second premolars or molars), screwdriver shaped incisors, microdontia, radiculomegaly, and enamel hypoplasia. Eruption was usually normal. Dental caries was associated with enamel defects, a high sugar/carbohydrate soft food diet, poor oral hygiene and dry mouth. Cephalometric analysis revealed mid-face hypoplasia, a small retroposed mandible and hypo-development of the skull.

**Conclusion:**

CS patients may have associated oro-dental features, some of which may be more frequent in CS children – some of them being described for the first time in this paper (agenesis of second permanent molars and radiculomegaly). The high susceptibility to rampant caries is related to a combination of factors as well as enamel developmental defects. Specific attention to these anomalies may contribute to diagnosis and help plan management.

## Background

Cockayne Syndrome CS (Type A - CSA or Type I - OMIM #216400); type B - CSB or Type II - OMIM #133540) is a rare autosomal recessive neurological disease caused by defects in DNA repair via nucleotide excision repair (NER), a molecular mechanism of disease shared also by Xeroderma Pigmentosum (XP) and Trichothiodystrophy (TTD) [1-3]. The incidence in Western Europe has been recently evaluated as 2.7 per million [4]. The main clinical features of all types are a progressive cachectic dwarfism, progressive neurological degeneration with cerebral leukodystrophy, microcephaly, progressive pigmentary retinopathy, sensorineural deafness and photosensitivity [5]. CS Type A (Type I) is defined as the classical milder form of the syndrome whereas CS Type B (Type II) is the early onset severe form, which can lead to early death. Cerebro-oculo-facio-skeletal syndrome (COFS) is a more severe prenatal form of CS with clinical expression similar to type II. CS Type III has mild symptoms and onset in late childhood. Different severity groups have however been described and renamed recently: severe, moderate and mild CS. Mean age of death is 5, 16 and 30 years in these groups, respectively. Very severe cases with prenatal onset and very mild cases with adult-onset have also been identified at both ends of the clinical spectrum [6,7]. CSA is caused by mutations in the excision-repair, cross-complementing group 8 gene (*ERCC8*) at 5q12 and CSB by mutations in the excision-repair, cross-complementing group 6 gene (*ERCC6*) at 10q11. There are no reported genotype/phenotype correlations [8-10]. Other genes - *XPB (ERCC3)*, *XPD (ERCC2)*, *XPG (ERCC5), XPF (ERCC4)* involved in XP are causative in patients presenting with a combination of XP and CS type B (II) [11-13].

Craniofacial anomalies and dysmorphism associated with CS are partially described in the literature. Microcephaly (a small head) with retrognathia (a distally placed mandible), prominence of the facial cheekbones, micrognathia (small lower jaw) [14] have been reported. High arched palate, atrophy of the alveolar process, condylar dysplasia, absence of some permanent teeth and short roots have been described [5,15-24]. Oro-dental features like delayed deciduous tooth eruption, malocclusion, absent/hypoplastic teeth were also mentioned by Nance and Berry [5] but with no detailed analysis. Dental anomalies including dental caries, are considered to be a minor diagnostic feature in this milestone paper [5] together with photosensitivity, progressive retinitis pigmentosa, and deafness.

The aim of the present study was to investigate the oro-dental and cranio-facial findings in a series of CS 17 patients from 15 families with a confirmed diagnosis of CS, to ascertain their relevance to the phenotype and their variability, and to provide quantitative data of cephalometric analyses, to assess their usefulness for clinical diagnosis, and to ascertain whether there are possible genotype/phenotype correlations.

## Methods

### Patients

A total of 17 CS patients (in 15 families), from France, the UK, the Netherlands, Switzerland and Morocco participated in this sub-study of the PHRC 2005 “Clinical and molecular study of Cockayne syndrome”.

Families gave informed consent. All clinical and molecular studies were approved by the Local Ethics Committee of the Strasbourg University Hospital. For each patient, the diagnosis of CS was confirmed using cellular (defect in TCR pathway) and molecular (identified mutations in *CSA* or *CSB*) analyses. Mutations have been previously reported [25]).

Patients were examined clinically by 2 different dental surgeons in the Reference Centre for Oro-dental Manifestations of Rare Diseases, Pôle de Médecine et Chirurgie Bucco-Dentaires, Hôpitaux Universitaires, Strasbourg, France. Inter-investigator agreement was assessed through comparison of cases and discussion. The oro-dental findings were documented using the D[4]/phenodent registry: a **D**iagnosing **D**ental **D**efects **D**atabase (see http://www.phenodent.org, to access assessment form). This registry allows standardization of data collection and, therefore, assists in oro-dental phenotyping. It facilitates providing clinical care to patients, a basis for genotype/oro-dental phenotype correlations, and sharing of data and clinical material between clinicians.

Computed tomography (CT) examination of the head was acquired for each patient within the Department of Radiology, University Hospital, Strasbourg, by a high resolution spiral equipment (SOMATOM Sensation 16® scanner, Siemens® Medical Solutions, Erlangen, Germany). CT scans were also performed to assess the neuroimaging of the brain in order to update and improve the description of the neuroimaging characteristics of the different clinical subtypes of CS. Sedation was used in some patients [26].

Axial images of 1mm thickness were made with 0.7 intervals and a field of view of 220 mm.

All the axial CT data were reformatted to generate images parallel to the Frankfurt reference plane. Lateral and frontal cranial cephalometric projections were obtained from the 3D MIP reconstruction of the skull. Panoramic and cross-sectional images of the maxilla and mandible were generated for examination of the teeth and periodontium.

Cephalometric analyses were performed in *norma lateralis* and *norma frontalis* from the CT-cranial projections [27]. The method, landmarks, reference lines, and measurements are described in Additional files 1 and 2 and illustrated in Additional files 3 and 4. Dental radiographic examination consisted of the panoramic and cross-sectional reconstructions. Dental abnormalities of number, shape, size, structure, eruption… such as agenesis, impacted teeth, were then analyzed for each patient.

## Results

Seventeen patients aged between 1 to 28 years from 15 families and diagnosed clinically with CS were examined between September 2006 and October 2009 during the PHRC « Clinical and molecular study of Cockayne syndrome ». The clinical diagnosis was confirmed with the discovery of mutations in *CSA* gene for 5 patients and *CSB* gene for 10 patients. For 2 brothers (patients 12 and 14) no genomic mutations were found also there was total absence of *CSA* mRNA (Table 1).

**Table 1:** Genotype and oro-dental features encountered in our cohort of 17 CS patients

**Patient**	**Age (y.m)**	**Type**	**Gene**	**Mutation**	**Orodental anomalies**							**Crowding**	**Caries dmft / DMFT**	**Perio**	**Oral Hygiene**	**Dental treatment**	**FD**	**Para**
					**Number of teeth**	**Shape**	**Size**	**Structure**		**Eruption**								
								**Enamel**	**Dentin**	**pd**	**PD**							
**1**	1.8	Severe (II)	*CSB*	c.2167C>T	Ø	Ø	microdontia	opacities	Ø	late	Ø	+	0 / Ø	normal	good AB	First visit	MB	Ø
CS3LE				c.2578_80delCTG														
**2**	1.9	Very severe COFS	*CSB*	c.2612T>C c.3513dupT	Ø	Ø	microdontia	opacities	Ø	Ø	Ø	Ø	0 / Ø	normal	absent NB	VI	MB	brux
CS881VI																		
**3**	2.3	Severe (II)	*CSB*	c.1954C>T	Ø	Ø	microdontia	hypoplasia	Ø	Ø	Ø	Ø	0 / Ø	normal	good AB	VI	MB	brux
08STR4				c.1954C>T														
**4**	2.5	Very severe COFS	*CSB*	c.2960T>C	Ø	Ø	microdontia	hypoplasia	Ø	Ø	Ø	Ø	0 / Ø	normal	absent NB	First visit	MB R	Ø
CS797VI				c.2254A>G														
**5**	2.9	Severe (II)	*CSB*		Ø	Ø	Ø	hypoplasia	Ø	Ø	Ø	Ø	20 / Ø	gingivitis	deficient NB	First visit	MB R	Ø
**6**	3.7	Severe (II)	*CSB*	c.3862C>T	Ø	Ø	Ø	hypoplasia	Ø	Ø	Ø	Ø	20 / Ø	normal	absent NB	VI No treatment	MB R	brux
CS817VI																		
**7**	4.11	Moderate (I)	*CSB*	c.2830-2A>G	Ø	Ø	microdontia	hypoplasia	Ø	Ø	Ø	Ø	0 / Ø	normal	good AB	VI	MB	Ø
				c.3983dupA														
**8**	6.7	Moderate (I)	*CSB*	c.653-2A>G	agenesis 12, 15, 25,35,45	Ø	microdont 22	Ø	Ø	Ø	Ø	+++	2 / Ø	normal	good AB	VI no treatment Ortho ass	MB	Ø
AEN74																		
**9**	7.11	Moderate (I)	*CSA*	c.618–1G>A	Ø	Ø	Ø	hypoplasia	Ø	early	early	++	5 / 0	gingivitis	deficient AB	VI LA	MB D R	Ø
CS794VI																		
**10**	8.1	Moderate (I)	*CSA*	c.582G>C	agenesis 35,17,37, 47	11, 21 shovel 12,22 atyp	taurodontism	opacities	Ø	Ø	Ø	Ø	2 / Ø	gingivitis	deficient AB	VI	MB D	Ø
CS655VI				c.70dupA														
**11**	9.1	Moderate (I)	*CSA*	c.797A>G	Ø	11, 21 screw d.	Ø	opacities	Ø	early	early	Ø	0 / 0	gingivitis	deficient AB	VI Fissure sealants under CSE	MB D	Ø
CS861VI				c.843+5G>C														
**12**	9.5	Moderate (I)	*CSA*	r.0 (no mRNA full length)	Ø	12, 22 cingulum	globular 2nd premolar	opacities	Ø	Ø	Ø	+++	16 / 4	gingivitis	deficient NB	2GA	MB R	Ø
CS466VI																		
**13**	14.4	Moderate (I)	*CSA*	c.797A>G	Ø	Ø	Ø	hypoplasia	Ø	Ø	Ø	Ø	Ø / 0	gingivitis	deficient AB	1GA Primary dentition	MB D	Ø
08STR2																		
**14**	14.6	Moderate (I)	*CSA*	r.0 (no mRNA full length)	Ø	Ø	Ø	hypoplasia	Ø	Ø	Ø	?	Ø / 28	gingivitis	deficient NB	4GA	MB R	Ø
CS466VI_2																		
Brother of CS466VI																		
**15**	15.10	Mild (III)	*CSB*	c.1913A>G	Ø	11, 21 screw d.	Ø	opacities	Ø	Ø	Ø	+++	Ø / 0	gingivitis	deficient AB	VI, LA Ortho ass	MB	Ø
CS543VI				c.2247delT														
**16**	16.5	Moderate (I)	*CSA*	c.797A>G	Ø	Ø	radiculo- megaly	hypoplasia	Ø	Ø	Ø	+	Ø / 2	gingivitis	deficient AB	1GA	MB D	Ø
08STR2_2																		
Brother of 08STR2																		
**17**	28.5	Mild (III)	*CSB*	c.3778+2T>G	Ø	11, 21 shovel	taurodontism	pits	Pulp Calcif	Ø	Ø	+++	Ø / 13	gingivitis	deficient AB	1GA Ortho ttt	MB	Ø
CS823VI				c.2203C>T														

The patient identification such as CS3LE and genotype was previously described in [25].

pd: primary dentition ; PD: permanent dentition; screw d : screw driver ; dmft/DMFT : Decayed Missing and Filled Teeth for primary dentition (dmft) and permanent dentition (DMFT) teeth ; Perio: periodontium; The level of oral hygiene was correlated to the abundance of dental plaque and related to the brushing habits (from assisted AB to absent NB); Dental treatment was either: the child’s first visit to the dentist First, regular visit to the dentist VI, dental treatment in the chair under local anesthesia LA, dental procedures under conscious (inhalation) MEOPA/nitrous oxide sedation (CSE), dental treatment under general anesthesia GA, previous orthodontic assessment or treatment Ortho ass or ttt; FD: functional defects, MB: mixed breathing (mouth and nose), D: deglutition or swallowing defect, R: reflux, Para: parafunctions, brux: bruxism ; Ø none ; + present.

Seven patients were in the primary dentition stage, 5 patients had mixed dentition and 5 patients had a permanent dentition.

**Figure 1:**
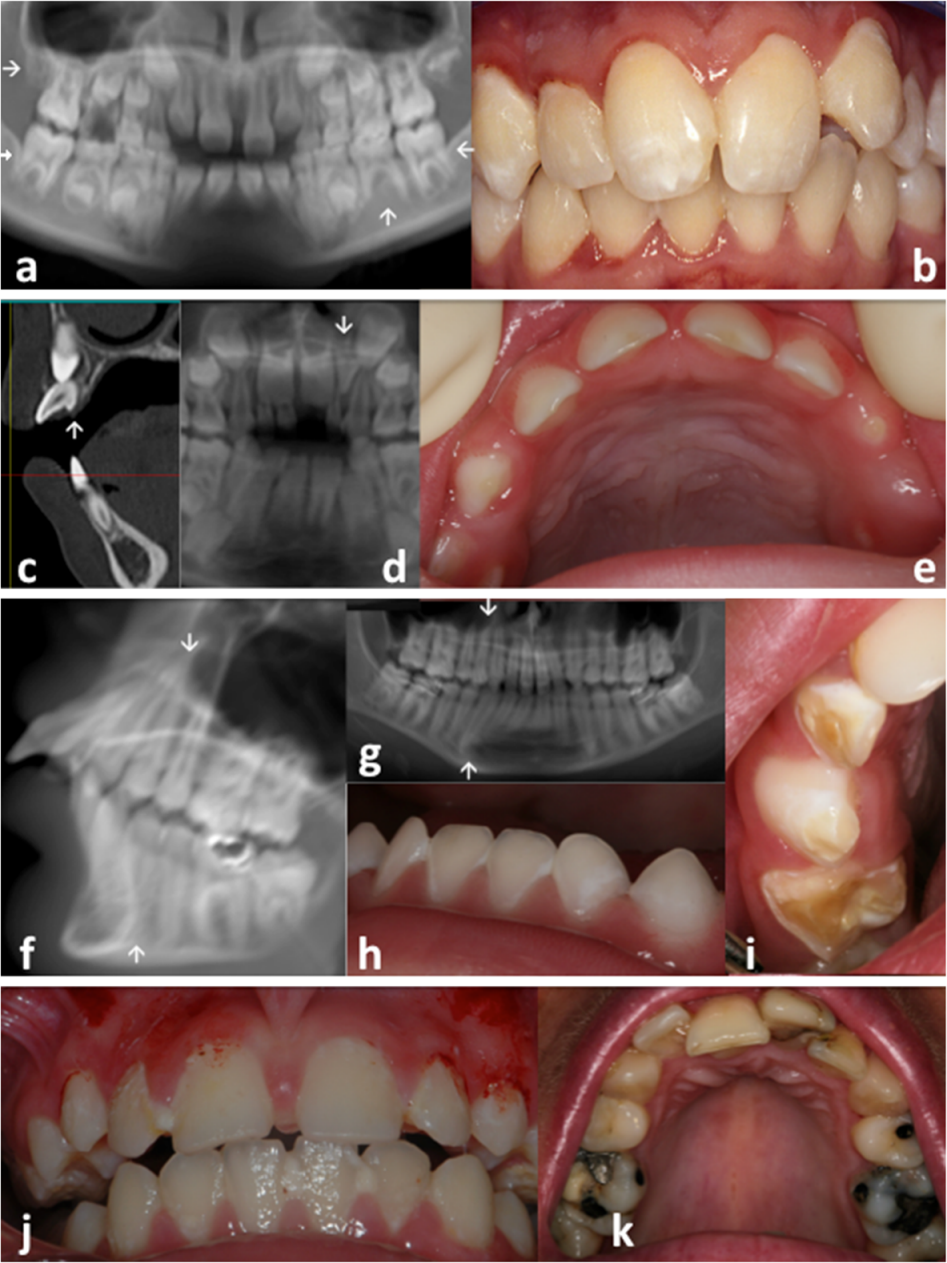
**Oro-dental features encountered in patients presenting with Cockayne syndrome(see also Table **1**)**. Anomalies of **tooth number** (**a**) - three congenitally missing second permanent molars (teeth 17, 37, 47) and the lower inferior left premolar (35) (arrow) in patient 10; **tooth shape** (**b**) - screwdriver shape incisors (teeth 11, 21 in patient 15); (**c**) hyper developed cingulum on the permanent upper lateral incisors (patient 12); **tooth size** (**d**) - permanent dentition with a microdont conical upper left lateral incisor (arrow) (tooth 22 in patient 8; the 12 is congenitally missing); (**e**) with microdontia in the primary dentition (patient 4, notice the diastemata separating the smaller primary teeth); (**a**) taurodontic upper permanent first molars in patient 10; radiculomegaly of canines, premolars and molars (**f**,**g**, patient 16) – (**i**) **tooth structure** with enamel hypoplasia (**h** in patient 2, **i** in patient 6) in the primary dentition. Dental plaque and biofilm subsequent to poor oral hygiene as well as gingivitis were seen in patient 11 (**j**). Dental crowding was visible in (**k**) for patient 17. ((**a**) patient 10; (**b**) patient 15; (**c**) patient 12; (**d**) patient 8; (**e**) patient 4; (**f;g**) patient 16; (**h**) patient 2; (**i**) patient 6; (**j**) patient 11; (**k**) patient 17).

A summary of all major findings related to oro-dental anomalies is provided in Table 1. The oro-dental anomalies recorded displayed extreme heterogeneity and variability both in the type of anomalies and their severity; some of these anomalies are being described for the first time in this paper (agenesis of second permanent molars and radiculomegaly). Dental findings can be classified into:

· **Anomalies of tooth number:** hypodontia (fewer than 6 missing permanent teeth excluding third molars) which was discovered on radiographs for 2 (2/17) individuals. The missing teeth were the upper right lateral incisor (5,88%) (12), the second premolars, as well as three of the second molars (5,88%)(Figure 1a).

The prevalence, in the European population, of hypodontia is 5,5%; of agenesis of maxillary lateral incisors is 1.55 - 1.78%; of second upper premolars is 1.39 - 1.61% and permanent second molars is 0.03 - 0.06% [28].

**Anomalies of tooth size and shape:** Shovel (incisors whose lingual surfaces are scooped) or screw-driver-shaped (the teeth are narrower at the incisal edge) upper permanent central incisors were the most striking abnormal features (Figure 1b) affecting 4 of 17 individuals. Other anomalies included abnormal crown and root form of the upper lateral incisors (patient 10) (1/17), hyper developed cingula of the lateral incisors (Figure 1c) and globular premolars. Five (5/17) children had microdont primary teeth (Figure 1e). A microdont upper permanent lateral incisor (22) opposite a missing contra-lateral tooth (12) was seen in patient 8 (1/17) (Figure 1d). Taurodontic first upper permanent molars (patients 10,17) (Figure 1a) and radiculomegaly on canines, premolars and molars (patient 16) (Figure 1f,g) were also observed on radiographs.

**Anomalies of tooth structure:** generalized demarcated enamel opacities and hypoplasia and pits were seen in 16 out of 17 patients both in the primary (Figure 1e,h,i) and permanent dentitions (Figure 1b,j,k). Intrapulpal calcifications were discovered in one patient (17). Hypoplasia in the primary dentition was clearly visible in very young patients and affected surfaces of teeth rarely exposed to decay confirming that the enamel defect occurred prior to secondary carious lesions (4 (Figure 1e), 2 (Figure 1h), 6 (Figure 1i), 1, 3, 7, 5). For example, the patient (6) illustrated in Figure 1i was never mouth fed.

**Anomalies of tooth eruption/exfoliation:** Eruption was usually normal. Two patients however showed early eruption of the primary and permanent teeth. A child of 20 months (1/17) had delayed eruption with only 4 erupted primary teeth.

**Dental caries** was present in 9/17 patients, with some individuals being severely affected with a dmft (Decayed, Missing, Filled Teeth) score ranking between 16 and 20 for the primary dentition (out of 20) and a DMFT (Decayed, Missing, Filled Teeth) score between 13 (Figure 1k) and 28 for the permanent dentition (out of 32). 47% of patients were caries free. In preschool children, in the primary dentition, the two patients showing the higher dmft index were both affected by a moderate form of the disease, and presented with enamel hypoplasia. They both had poor or absent oral hygiene and both suffered from gastro-oesophageal reflux. It is interesting to notice that most of the patients with a high caries rate were suffering from gastro-oesophageal reflux. Most patients needed assistance to maintain oral hygiene. For patient 5 the cleaning of the oral cavity was performed solely using gauze compress. Assisted brushing became more difficult as patients grew older and subsequently patients had gingivitis associated with dental plaque and poor or deficient oral hygiene habits (Figure 1b,j). The marginal gingiva had a thin biotype (Figure 1e).

**Occlusion:** Crowding and tooth malposition were prevalent in the mixed and the permanent dentitions (Figure 1k) (6 of all patients).

**Functional defects** included mixed breathing (mouth and nose) leading to dry mouth, atypical or immature deglutition (normal under 8–10 years of age) and parafunction such as bruxism (3/17 patients). Gastro-oesophageal reflux and vomiting were present in 35% of patients (6 out of 17 patients). Feeding was always difficult, leading to failure to thrive, even with soft diet and gastrostomy.

Craniofacial skeletal anomalies

For all patients except one it was not possible to use standard panoramic radiograph. Some patients were sedated for the head CT examination [26]. Cephalometric analysis (for skeletal dysmorphology) was performed on only 9 patients from 8 families aged between 6.7 and 28.5 years to allow comparison with known standards (Table 2). Seven patients were below 4 years of age. One patient had no radiographic data. The *norma lateralis* analysis showed a typical profile characterized by an Angle Class II (in Angle Class II the upper jaw (maxilla) can be too far forward or, more usually as it is the case here, the lower jaw (mandible) is too far back) skeletal growth pattern with posterior rotation of the mandible and retrognathism (a small distally placed mandible) (Table 2, Additional File 3). Measurements in *norma frontalis* suggested a general tendency to overall mid-face and skull hypoplasia (Table 2, Additional file 4).

**Table 2:** **Results of the cephalometric analysis in *norma lateralis and frontalis***

***Measurement***	**Patient 8**		**Patient 9**		**Patient 10**		**Patient 11**		**Patient 12**		**Patient 14**		**Patient 15**		**Patient 16**		**Patient 17**		**CS**
	**(6.7)**		**(7.11)**		**(8.1)**		**(9.1)**		**(9.5)**		**(14.6)**		**(15.10)**		**(16.5)**		**(28.5)**		
***Norma lateralis***																			
**Typology**																			
**Facial axis (°) (1)**	81.0	(89.5; 3.8)	79.6	(89.6 ;4.0)	88.0	(89.5; 3.6)	89.0	(89.6; 4.1)	76.0	(89.3; 4.3)	76.0	(89.4; 4.3)	74.0	(89.2; 4.5)	96.0	(88.9; 4.6)	80.0	(89.0; 4.0)	**↑**
**Facial depth (°) (2)**	73.0	(83.2; 3.2)	84.1	(83.8 ;2.8)	81.0	(82.1; 3.3)	80.0	(84.3; 3.0)			74.0	(83.3; 3.7)	67.0	(82.9; 4.5)	75.0	(82.5; 3.9)	78.0	(86.0; 2.5)	↓
**Lower facial height (°) (3)**	56.0	(47.0; 4.0)	53.2	(47.0 ;4.0)	43.0	(47.0; 4.0)	41.0	(47.0; 4.0)	58.0	(47.0; 4.0)	50.0	(47.0; 4.0)	51.0	(47.0; 4.0)	47.0	(47.0; 4.0)	50.0	(47.0; 4.0)	**N**
**Mandibular arc (°) (4)**	31.0	(26.0; 3.5)	20.1	(26.0 ;3.5)	26.0	(26.0; 3.5)	52.0	(26.0; 3.5)	13.0	(26.0; 3.5)	18.0	(28.0; 3.5)	22.0	(29.0; 3.5)	16.0	(29.0; 3.5)	22.0	(30.0; 3.5)	↓
**FMA (°) (5)**	45.0	(29.4; 4.5)	38.8	(28.6 ;3.8)	36.0	(29.4; 4.8)					50.0	(27.7; 5.8)	44.0	(28.5; 6.2)	36.0	(28.7; 5.2)	41.0	(25.8; 3.0)	**↑**
**AFH (mm) (6)**	50.0	(57.1; 3.1)	76.4	(53.3;3.3)	46.0	(61.8; 3.6)	46.0	(60.2; 3.6)	50.0	(62.8; 3.9)	52.0	(70.7; 5.5)	51.0	(73.3; 5.8)	49.0	(76.1; 5.6)	58.0	(67.2; 4.3)	↓
**PFH (mm) (7)**	22.0	(37.9; 3.7)	28.5	(33.6 ;2.7)	25.0	(42.2; 3.4)	23.0	(41.2; 3.5)	25.0	(43.4; 3.3)	19.0	(51.4; 4.6)	22.0	(51.4; 4.6)	26.0	(54.3; 4.1)	30.0	(49.6; 3.9)	↓
**FHI (%)**	0.4	(0.6)	0.37	(0.6)	0.5	(0.6)	0.5	(0.6)	0.5	(0.6)	0.3	(0.7)	0.4	(0.7)	0.5	(0.7)	0.5	(0.7)	↓
**Skelatal analysis**																			
**Convexity (mm) (8)**	10.0	(4.5; 2.2)	11.9	(4.1 ;2.4)	5.0	(4.4; 2.5)	12.0	(3.6; 2.7)	7.0	(3.8; 2.3)	11.5	(2.8; 2.6)	9.0	(2.8; 2.8)	9.0	(2.6; 3.4)	8.5	(1.7; 2.9)	**↑**
**ANB (°) (9)**	15.0	(4.7; 2.2)	10.9	(4.6 ;2.4)	8.0	(4.8; 2.2)	14.0	(4.0; 2.6)	10.0	(4.2; 1.9)	13.0	(3.4; 2.0)	11.0	(3.3; 2.1)	11.0	(3.2; 2.3)	10.0	(2.6; 2.4)	**↑**
**Denture analysis**																			
**i to APg (mm) (10)**	4.0	(−0.5; 2.7)	7.2	(0.9 ;2.4)	5.0	(1.1; 2.5)	8.0	(1.6; 2.7)	6.0	(1.8; 2.4)			12.0	(1.9; 2.6)	9.0	(2.8; 2.9)	8.0	(0.8; 2.8)	**↑**
**i to APg (°) (11)**	22.0	(15; 7.2)	25.1	(20.7 ;6.3)	44.0	(20.8; 5.2)	32.0	(22.1; 6.2)	24.0	(22.1; 4.8)			35.0	(23.8; 5.4)	37.0	(25.2; 4.9)	33.0	(21.8; 7.3)	**↑**
**FMIA (°) (12)**	48.0	(64.8; 7.5)	59.1	(58.0 ;9.1)	45.0	(56.4; 7.7)							36.0	(55.9; 8.1)	42.0	(55.6; 8.2)	43.0	(59.0; 10.7)	↓
**IMPA (°) (13)**	87.0	(87.9; 7.2)	82.1	(93.1 ;7.0)	99.0	(94.0; 5.7)	105.0	(93.9; 7.2)	81.0	(94.7; 5.7)			100.0	(94.8; 7.2)	102.0	(95.3; 6.6)	96.0	(92.1; 9.0)	**N**
**i/I (°) (14)**	128.0	(142.2; 14.2)	110.8	(127.2 ;10.2)	110.0	(128.1; 11.2)	107.0	(125.5; 9.7)	117.0	(126.3; 9.2)			114.0	(129.2; 10.1)	103.0	(126.6; 10.0)	119.0	(133.6; 13.0)	↓
***Norma frontalis***																			
**Cranial width (mm) (15)**	103.0	(135.7; 5.4)	180.9	(140.2; 4.3)	137.0	(140.2; 4.3)	132.0	(136.5; 5.6)	103.0	(140.2; 4.3)	116.0	(143.2; 4.7)	117.0	(143.2; 4.6)	120.0	(143.2; 4.6)	132.0	(139.1; 5.5)	↓
**Bifrontotemporale width (mm) (16)**	69.0	(92.3; 4.9)	129.3	(95.4; 3.0)	85.0	(95.4; 3.0)	92.0	(93.9; 5.5)	72.0	(95.4; 3.0)	81.0	(100.3; 3.6)	79.0	(100.3; 3.6)	92.0	(100.3; 3.6)	93.0	(96.5; 4.5)	↓
**Bizygomatic width (mm) (17)**	85.0	(109.4; 3.2)	128.4	(114.2; 4.2)	101.0	(114.2; 4.2)	105.0	(112.9; 3.4)	94.0	(114.2; 4.2)	100.0	(128.1; 4.4)	101.0	(128.1; 4.4)	105.0	(128.1; 4.4)	107.0	(122.8; 3.5)	↓
**Nasal width (mm) (18)**	22.0	(26.2; 1.6)	40.6	(29.5; 2.0)	20.0	(29.5; 2.0)	22.0	(27.5; 1.6)	23.0	(29.5; 2.0)	26.0	(34.3; 2.6)	23.0	(34.3; 2.6)	24.0	(34.3; 2.6)	25.0	(30.5; 1.5)	↓
**Bigonial width (mm) (19)**	65.0	(80.4; 3.8)	113.9	(83.0; 3.5)	77.0	(83.0; 3.5)	85.0	(83.5; 3.1)	66.0	(83.0; 3.5)	76.0	(94.6; 4.6)	75.0	(94.6; 4.6)	78.0	(94.6; 4.6)	89.0	(91.5; 3.1)	↓

For each measurement, the age-corresponding cephalometric standards are given (Mean; S.D.) **N** : Normal values. **↑** : Increased values. **↓** : Reduced values.

In patient 8 angle ANB is 15 ° compared to standard angle measure of 4.7° and confirms the skeletal class II. The standard deviation is 2.2. (x) indicates the measure (in distance or degree for angles) area visible on Additional files 3 and 4.

### Access to dental treatment

For 3 patients below 3 years of age, participation in the research program was their first opportunity to have an oral cavity examination and for their parents to discuss the dental findings with the dentist and to learn the importance of regular home oral hygiene and dental preventive care. Two patients presenting with all decayed primary teeth had not yet received dental treatment. Four patients (moderate to mild type) had treatment in private dental practices (one had fissure sealants done under conscious sedation, and two had local anaesthesia). Five patients had received general anaesthesia for dental treatment (two of them numerous time from 2 to 4 years of age; patient 12 had 12 primary and 4 permanent teeth extracted and patient 14 had 12 primary and 22 permanent teeth extracted). One patient (mild type CS) had orthodontic treatment.

## Discussion

This Cockayne syndrome patients’ cohort displayed all the oro-dental anomalies described previously in the literature (Additional file 5[5,7,9,14-20,22-24,29-42]).

Various anomalies of the number, shape, size, structure and eruption of teeth demonstrated disturbance of tooth development. Agenesis of upper lateral incisors (teeth 12,22) and second premolars (teeth 15,25,35,45) is relatively common in the general population and teeth 12 and 22 are the most frequent congenitally absent teeth [43,44]. The prevalence, in the European population, of hypodontia is 5,5%; of agenesis of maxillary lateral incisors is 1.55 - 1.78%; of second upper premolars is 1.39 - 1.61% and permanent second molars is 0.03–0.06% [28]. Of special interest was the rare agenesis of the second permanent molars and the radiculomegaly noted. Molar agenesis has been associated with *PAX9* mutations [45] and recently with desmoplakin gene (*DSP*) mutations in Carvajal/Naxos syndrome [46]. Radiculomegaly has also been described in Oculofaciocardiodental (OFCD) syndrome due to mutations in *BCOR* gene [47] a transcriptional corepressor through the proto-oncoprotein, BCL6.

Developmental dental anomalies result from factors interfering with odontogenesis and with dental hard tissue mineralization (of enamel and dentine) are not subject to changes related to age or aging, they reflect interference with specific genetic and developmental biological processes [48,49], relating to the embryonic origin of tooth forming cells which are responsible for tooth formation, the types and arrangement of teeth, their defined location, and specific pattern of morphogenesis, histogenesis, and of terminal differentiation of odontoblasts and ameloblasts, leading to dentine and enamel matrix synthesis and their mineralization. This predetermined mechanism is similar for periodontium formation and the eruption of teeth [50-53].

The dental developmental anomalies described in CS might substantiate the hypothesis of a transcriptional defect in the pathogenesis of developmental anomalies observed in CS [54].

Dental caries, an acquired, multifactorial, infectious disease, was stated minor associated feature in CS [5], and was found to occur secondarily, in pre-existing enamel developmental defects (opacities, hypoplasia). Its’ initiation and progress can be accelerated by soft high sugar/carbohydrate diet, poor oral hygiene and dry mouth (reduced salivary flow). Gastro-oesophageal reflux and vomiting may induce enamel erosion of the palatal surfaces of mainly the incisors, but under normal circumstances rarely results in dental caries. The pre-existing enamel lesions could be a factor in the speed and the extent of dental caries progression. This is the first study of CS patients, which analyses the multifactorial origin of this feature. Natale [7] stated that dental caries occurred frequently in CS as it does in the normal population, but that no correlation with CS severity was found.

No cephalometric data explaining the craniofacial dysmorphism in CS was found in the literature. The use of lateral cephalograms in the differential diagnosis of jaw and craniofacial anomalies and treatment planning, has become generally accepted as the standard in orthodontics [55,56]. As a result of further innovation in X-ray technology, digital radiology is now used regularly for dental and orthodontic diagnosis [57]. With complex anomalies, such as CS, it may be preferable to use modern imaging methods of craniofacial imaging such as cone-beam computed tomography (CBCT) or on occasion computed tomography (CT) to obtain sufficient detailed information for diagnosis and treatment planning. This can also be used in the assessment of progress of treatment. In this study to avoid multiple imaging with the ensuing increased exposure to radiation, it was considered reasonable to use existing CT datasets to obtain virtual frontal and lateral skull images and evaluate them with the aid of computers. In programs for processing of Dicom datasets, it is possible to calculate virtual summation images from the three-dimensional volume datasets that closely resemble conventional X-ray images. Recent studies demonstrated conventional frontal and lateral cephalograms were not necessary, as they could be created from the CT dataset with comparable evaluative accuracy [58,59]. Thus we performed cephalometric analyses in *norma lateralis* and *norma frontalis* from the CT-cranial projections. Measurements in *norma frontalis* suggested a general tendency to an hypo-development of the face and the skull. The *norma lateralis* showed a typical profile characterized by Angle skeletal class II with posterior rotation of a smaller mandible, and retrognathia. Facial morphology and therefore also dysmorphology change markedly with age; however it was not possible to observe evolving changes in dysmorphology in the small sample of 9 patients aged between 6.7 and 28.5 years on which cephalometric analyses were performed.

Some affected patients (6/17) had never visited a dentist or received previous oral health care advise or treatment. Two were suffering from extensive dental caries.

Patients with moderate to mild form of CS were able to accept a wide range of dental treatments, from simple preventive and dental caries control visits, to conventional restorations in the dental office under local anaesthesia or with conscious sedation, to more extensive restorative or surgical (tooth extraction) treatment under general anaesthesia. The treatment modalities were chosen taking into account the possible cooperation of the patient (age group, severity of the disease) and the extent of the existing oral pathology. There are reports of difficulties with general anaesthesia procedures in CS such as difficult airway and intubation management, and increased risks of gastric aspiration, later cachexia and accelerated aging issues [60,61]. A benefit/risk decision shared by all involved heath professionals caring for the patient and which is fully understood and agreed to by parents is necessary in order to undertake dental treatment under general anaesthesia. Emphasis should be placed on the importance of early preventive oral health measures, which if implemented should avoid the added burden of the need to use general anaesthesia for treatment in most cases.

No genotype/phenotype correlation related to craniofacial or oro-dental anomalies was detected in this patient cohort. Most of the studies published so far also describe variability in phenotype and no specific genotype/phenotype correlations [5,7,25].

## Conclusions

While this study was not able to conclude that there is a specific oro-facial and dental phenotype, which could assist in the diagnosis of CS, CS patients may have oro-facial and dental features a number of which are also present (with similar frequency) in the normal population. Two dental features are however described for the first time in this paper – these are agenesis of second molars and radiculomegaly. A high susceptibility to rampant dental caries as described in this study is also reported in a number of papers in the literature. Dental caries (which also occurs in a rampant form in most normal populations) is due to a conjunction of factors, for example a high carbohydrate soft diet, non-removal of the oral biofilm regularly and possibly here to hypoplastic enamel defects. Dental health education for parents and children should be emphasised and scheduled within the overall management of children suffering from CS. This health care advice should include oral hygiene advice, assisted brushing techniques, the appropriate use of topical fluoride and regular visits to the dentist. Prevention should commence as soon as primary teeth erupt. Oral hygiene should be maintained even when gastrostomy feeding is used.

Changes in jaw relationship, Angle Class II skeletal growth pattern with posterior rotation of a smaller mandible and retrognathism, which occur in CS, are also similar to those which occur in the normal population, but with a higher prevalence in CS. Due to the discrepancy between the size of the jaws and the teeth, malocclusion and crowding are frequent. Orthodontic treatment may be appropriate for children with CS (Type III) and possibly some children with CSA (Type I) where they are expected to live into or beyond the second decade. Reference centres for rare diseases play an instrumental role in the dissemination of knowledge and management of oro-facial and dental manifestations encountered in CS patients.

## Abbreviations

CS: Cockayne syndrome; NER: Nucleotide excision repair; XP: Xeroderma Pigmentosum; TTD: Trichothiodystrophy; COFS: Cerebro-oculo-facio-skeletal syndrome; CT: Computed tomography; CBCT: Cone-beam computed tomography;  : The international tooth numbering system indicates teeth by numbering as follows:;  : For the permanent dentition:;  : In the upper arch (maxilla) from the posterior of the right quadrant to the midline and from the midline to the posterior of the left quadrant. (ie. 18 - 11 & 21 - 28);  : In the lower arch (mandible) numbering commences in the lower left quadrant to the midline and midline to the posterior of the right quadrant (ie. 48 - 41 &31 - 38);  : For the primary (deciduous) dentition:;  : As shown above for the permanent teeth except that the teeth are indicated as for the maxilla 55 - 51 &61-65 and for the mandible 85 - 81 &71 - 75.

## Competing interest

The authors declare that they have no competing interests.

## Authors’ contributions

ABZ designed and coordinated the study, saw all patients, revised all the clinical and X-Ray data, and wrote the manuscript. MR contributed to the clinical work, seeing patients and collecting clinical data. Computer tomography examination was carried out by MK. MS reformated the axial CT data and analysed with MR the data. MR, RM ED performed the cephalometric analyses. MR, VAL, AZ, HD contributed towards the analysis of the data and revision of the manuscript. All authors read and approved the final manuscript. All contributors have read and approved the submission to the Journal.

## Authors’ information

Agnès Bloch-Zupan and Morgan Rousseaux should be both considered as first authors.

## Supplementary Material

Additional file 1 **Definition of selected landmarks used in the cephalometric analysis in*norma lateralis and frontalis.*** Click here for file

Additional file 2 **Measurement definitions and correspondence with additional files **3**and **4**.** For each definition, the number in (brackets) indicates the representation of the measurement in Additional files 3 and 4. Click here for file

Additional file 3 **3D MIP reconstruction of the skull (a) and cephalometric analysis in*norma lateralis*(b) of patient 8 (6.7 years) (See Table **2**).** The names and definitions of the landmarks and measured euclidien distances and angles are given in Additional files 1 and 2. Observe the direction of vertical growth of the lower jaw (angle 9 FMA) and retrognathia (diminished angle 2 Facial depth) or skeletal class II (Angle 11 ANB) can be seen. Click here for file

Additional file 4 **Cephalometric analysis in *norma frontalis*of patient 16 (16.5 years).** Correspondence of landmarks and measurements are detailed in Additional files 1 and 2 respectively. Reported to age related standards, transversal craniofacial hypodevelopment is patent). Click here for file

Additional file 5 **Literature review of craniofacial and oro-dental findings in CS.** The description of the anomalies appears as stated in the reviewed papers using the following wording: Mandibular micrognathia: Mandibular hypoplasia [15], Underdeveloped mandible [33], Retruded chin [19], Retruded small mandible [20]. Micrognathia: Small oral cavity [30], Agenesis: Congenitally absent of 14, 23, 24 [15], Congenitally absent mandibular second premolars [19], Absent/hypoplastic teeth [5], Macrodontia: Inappropriately large teeth [33], Microdontia: Very small teeth [20], Enamel defects: Opacities/hypoplasia (PHRC), Dark pigmented teeth [16], Discolored teeth [20], Hypoplasia ([15]; PHRC), Absent/hypoplastic teeth [5], Ectopic eruption: Ectopically erupted first molars and ectopically placed molars [19], Dental caries: Dental extractions [33], pt primary teeth, PT permanent teeth. Click here for file
