# Supplementary material for: A possible cranio-oro-facial phenotype in Cockayne syndrome
Source: Orphanet J Rare Dis. 2013 Jan 14;8:9. doi: 10.1186/1750-1172-8-9 (PMC3599377; doi:10.1186/1750-1172-8-9)
Supplement: Additional file 1 — Definition of selected landmarks used in the cephalometric analysis innorma lateralis and frontalis. [file 1750-1172-8-9-S1.doc]

**Additional file 1 : Landmark definitions**

| *Landmark* | *Definition* |
| --- | --- |
| *Norma lateralis* | |
| A | A POINT: the most posterior point on the curve of the maxilla between the anterior nasal spine and the supradentale |
| ANS | ANTERIOR NASAL SPINE: the tip of the median, sharp bony process of the maxilla at the lower margin of the anterior nasal opening |
| Ar | ARTICULARE : the point of intersection of the inferior cranial base surface and the averaged posterior surfaces of the mandibular condyles |
| B | B POINT : the point most posterior to a line from infradentale to pogonion on the anterior surface of symphyseal outilne of the mandible |
| Ba | BASION : the most inferior, posterior point on the anterior margin of foramen magnum |
| DC | DC POINT : the center of the neck of the condyle on the BaN line |
| Gn | GNATHION: the most anterior-inferior point on the contour of the bony chin symphysis  determined by bisecting the angle formed by the mandibular plane and the line through pogonion and nasion |
| Go | GONION : the most inferior and posterior point at the angle of the mandible |
| Me | MENTON : the most inferior point on the symphyseal outline |
| N | NASION : the junction of the frontonasal suture at the most posterior point on the curve at the bridge of the nose |
| Or | ORBITALE : the lowest point on the average of the right and left borders of the bony orbit |
| Pog | POGONION : the most anterior point on the contour of the bony chin. Determined by the tangent through nasion |
| PM | PROTUBERANCE MENTI : the point on the front border of symphysis between point B and Pog |
| Po | PORION : the midpoint of the line connecting the most superior point of the radiopacity generated by each of the two ear rods of the cephalostat |
| PNS | POSTERIOR NASAL SPINE: The most posterior point at the sagittal plane on the bony hard palate |
| Pt | PTERYGO-MAXILLARY FISSURE : the most posterior and superior point on the contour of the pterygo-maxillary fissure |
| S | SELLA TURCICA : the center of the pituitary fossa of the sphenoid bone |
| Xi | Xi POINT : constructed point corresponding to the geometric centre of the mandibular branch (Ricketts, 1961) |
| *Norma frontalis* | |
| Eu | EURION : the point at either end of the greatest diameter of the skull |
| Ft | FRONTOTEMPORALE : the most medial point on the temporal line of the frontal bone |
| Zy | ZYGION : the most lateral point of the zygomatic arch |
| NB | NASAL BREADTH : the greatest distance between the right and left lateral bony walls of the nasal cavity |
| Go | GONION : the most inferior and lateral point at the angle of the mandible |
