# Supplementary material for: A possible cranio-oro-facial phenotype in Cockayne syndrome
Source: Orphanet J Rare Dis. 2013 Jan 14;8:9. doi: 10.1186/1750-1172-8-9 (PMC3599377; doi:10.1186/1750-1172-8-9)
Supplement: Additional file 2 — Measurement definitions and correspondence with additional files 3and 4. For each definition, the number in (brackets) indicates the representation of the measurement in Additional files 3 and 4. [file 1750-1172-8-9-S2.doc]

**Additional file 2. Measurement definitions**

| *Measurement* | *Definition* |
| --- | --- |
| *Norma lateralis* |  |
| Facial axis (°) (1) | Angle posterior and inferior formed by lines constructed from nasion-basion (N-Ba) and gnathion-pterygomaxillary fissure (Gn-Pt) |
| Facial depth (°) (2) | Angle posterior and inferior formed by Frankfort plane (porion-orbitale) and line nasion-pogonion |
| Lower facial height (°) (3) | Angle anterior and superior formed by lines constructed from Xi-anterior nasal spine (ANS) and Xi-protuberance menti (PM) |
| Mandibular arch (°) (4) | Angle posterior and superior formed by lines constructed from Xi-DC and Xi-protuberance menti (PM) |
| Convexity (mm) (8) | Distance between A point and line nasion-pogonion |
| i to APg (mm) (10) | Distance between lower incisor incisal edge perpendicular to line A point-pogonion |
| i to APg (°) (11) | Angle posterior and inferior formed by lower incisor axis and line A point-pogonion |
| FMIA (°) (12) | Angle posterior and inferior formed by Frankfort plane and lower incisor axis |
| FMA (°) (5) | Angle anterior and superior formed by Frankfort plane and Mandibular plane (menton-gonial intersection) |
| IMPA (°) (13) | Angle posterior and superior formed by lower incisor axis and Mandibular plane |
| ANB (°) (9) | Angle formed by drawing a line from A point to nasion and from nasion to B point |
| i/I (°) (14) | Interincisal angle |
| AFH (mm) (6) | Distance between line anterior nasal spine (ANS)-posterior nasal spine (PNS) and menton |
| PFH (mm) (7) | Distance between articulare and gonion |
| FHI (%) | Facial height index corresponding to the ratio posterior facial height (PFH)/anterior facial height (AFH) |
| *Norma frontalis* |  |
| Cranial width (mm) (15) | Distance between right and left eurion points |
| Bifrontotemporale width (mm) (16) | Distance between right and left frontotemporale points |
| Bizygomatic width (mm) (17) | Distance between right and left zygion points |
| Nasal width (mm) (18) | The greatest distance between the right and left lateral bony walls of the nasal cavity |
| Bigonial width (mm) (19) | Distance between right and left gonion points |

For each definition, the number in (brackets) indicates the representation of the measurement in Additional files 3 and 4.
