# Supplementary figures and images for: A possible cranio-oro-facial phenotype in Cockayne syndrome
Source: Orphanet J Rare Dis. 2013 Jan 14;8:9. doi: 10.1186/1750-1172-8-9 (PMC3599377; doi:10.1186/1750-1172-8-9)

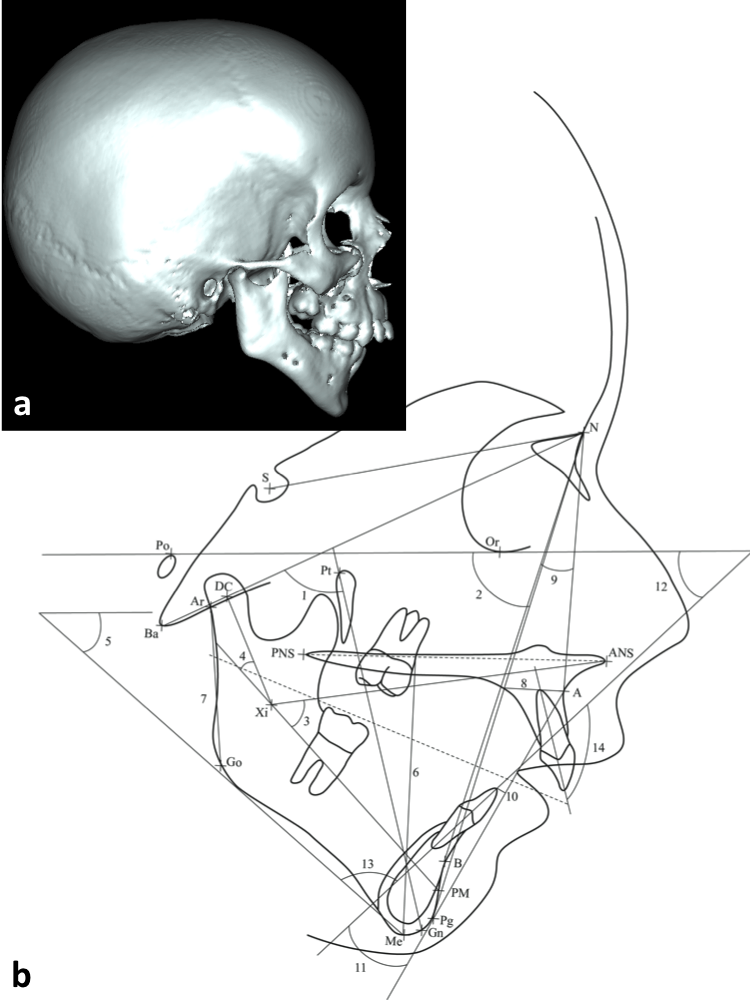

Supplement: Additional file 3 — 3D MIP reconstruction of the skull (a) and cephalometric analysis innorma lateralis(b) of patient 8 (6.7 years) (See Table 2). The names and definitions of the landmarks and measured euclidien distances and angles are given in Additional files 1 and 2. Observe the direction of vertical growth of the lower jaw (angle 9 FMA) and retrognathia (diminished angle 2 Facial depth) or skeletal class II (Angle 11 ANB) can be seen. [file 1750-1172-8-9-S3.tiff]

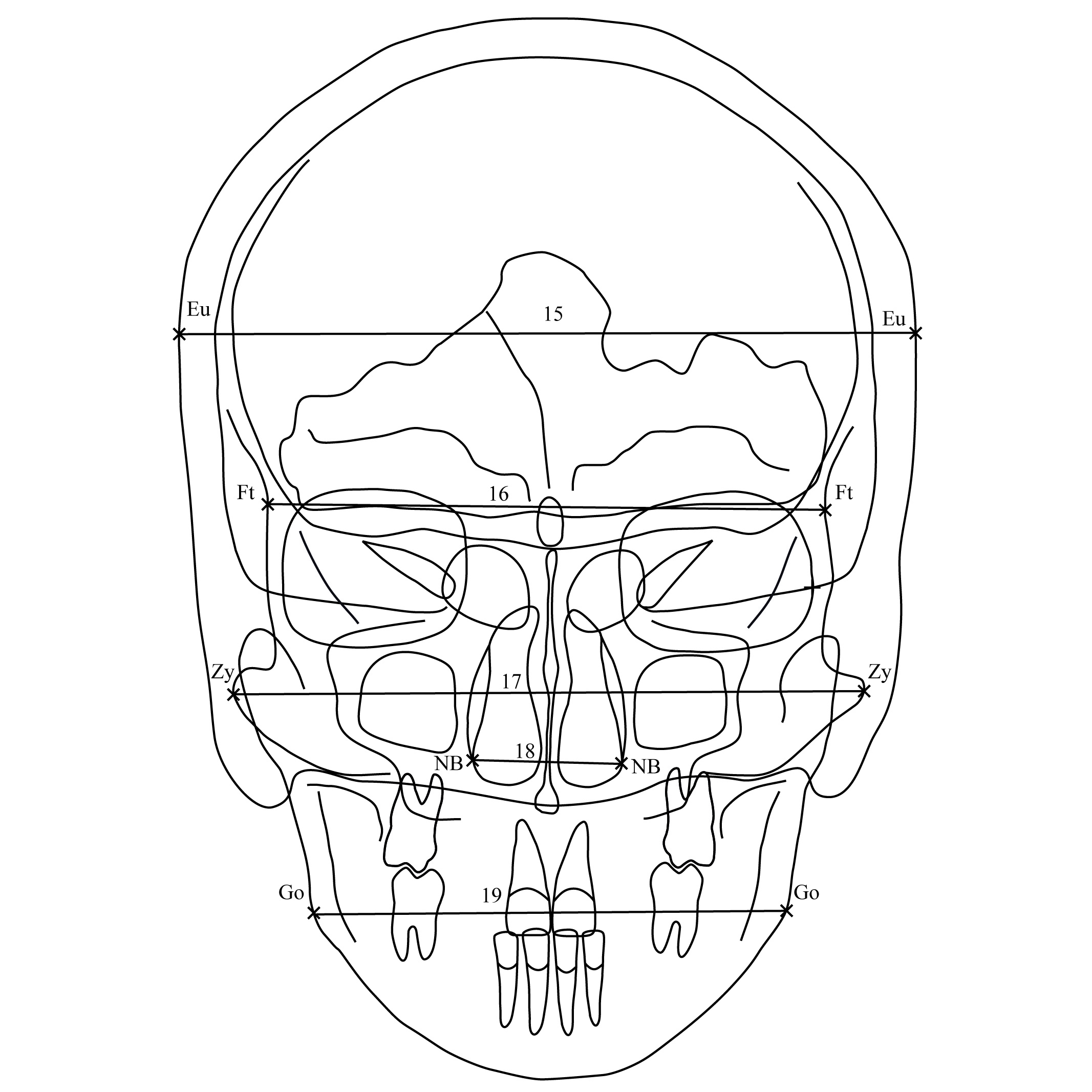

Supplement: Additional file 4 — Cephalometric analysis in norma frontalisof patient 16 (16.5 years). Correspondence of landmarks and measurements are detailed in Additional files 1 and 2 respectively. Reported to age related standards, transversal craniofacial hypodevelopment is patent). [file 1750-1172-8-9-S4.jpeg]
