# Supplementary material for: A possible cranio-oro-facial phenotype in Cockayne syndrome
Source: Orphanet J Rare Dis. 2013 Jan 14;8:9. doi: 10.1186/1750-1172-8-9 (PMC3599377; doi:10.1186/1750-1172-8-9)
Supplement: Additional file 5 — Literature review of craniofacial and oro-dental findings in CS. The description of the anomalies appears as stated in the reviewed papers using the following wording: Mandibular micrognathia: Mandibular hypoplasia [15], Underdeveloped mandible [33], Retruded chin [19], Retruded small mandible [20]. Micrognathia: Small oral cavity [30], Agenesis: Congenitally absent of 14, 23, 24 [15], Congenitally absent mandibular second premolars [19], Absent/hypoplastic teeth [5], Macrodontia: Inappropriately large teeth [33], Microdontia: Very small teeth [20], Enamel defects: Opacities/hypoplasia (PHRC), Dark pigmented teeth [16], Discolored teeth [20], Hypoplasia ([15]; PHRC), Absent/hypoplastic teeth [5], Ectopic eruption: Ectopically erupted first molars and ectopically placed molars [19], Dental caries: Dental extractions [33], pt primary teeth, PT permanent teeth. [file 1750-1172-8-9-S5.doc]

| **Sign or symptom** | **Author (publication year)** | | | | | | | | | | | | | | | | | | | | | | | | | | |
| --- | --- | --- | --- | --- | --- | --- | --- | --- | --- | --- | --- | --- | --- | --- | --- | --- | --- | --- | --- | --- | --- | --- | --- | --- | --- | --- | --- |
|  | Cockayne | Marie | Macdonald | Civantos | Lieberman | Guzzetta | Fujimoto | Rowlatt | Cotton | Schmickel | Scott-Emuakpor | Cook | Schneider | Boraz | Nance & Berry | Sorin | Dumić | Mallery | Meira | Hamamy | Tan | Arenas Sordo | Bertola | Falik-Zaccai | Natale | Wang | PHRC |
|  | (1936) | (1958) | (1960) | (1961) | (1961) | (1967) | (1969) | (1969) | (1970) | (1977) | (1977) | (1982) | (1983) | (1991) | (1992) | (1994) | (1995) | (1998) | (2000) | (2005) | (2005) | (2006) | (2006) | (2008) | (2011) | (2011) |  |
| **Craniofacial dysmorphism** |  |  |  |  |  |  |  |  |  |  |  |  |  |  |  |  |  |  |  |  |  |  |  |  |  |  |  |
| Facial dysmophism | X | X | X |  |  |  |  |  |  |  |  |  |  |  |  |  |  |  |  |  | X | X |  | X | X |  | X |
| Facial complex underdeveloped horizontally and vertically | X |  |  |  |  |  |  |  |  |  |  |  | X | X |  |  |  |  |  |  |  |  |  |  |  |  | X |
| Transversal hypo-development of the skull and face | X |  |  |  |  |  |  |  |  |  |  |  |  |  |  |  |  |  |  |  |  |  |  |  |  |  | X |
| Straight facial profile |  |  |  |  |  |  |  |  |  |  |  |  | X |  |  |  |  |  |  |  |  |  |  |  |  |  |  |
| Micrognathia |  |  |  |  |  |  |  |  |  |  |  |  |  | X |  |  |  |  |  |  | X |  |  |  |  |  | X |
| Sharp mandible |  |  |  |  |  |  |  |  |  |  |  |  |  |  |  |  |  |  |  |  |  |  |  |  |  | X |  |
| Mandibular micrognathia |  |  |  |  |  |  |  |  |  |  |  | X | X |  |  | X |  |  |  |  |  | X |  |  |  |  | X |
| Small oral aperture |  |  |  |  |  |  |  |  |  |  |  | X |  |  |  |  |  |  |  |  |  |  |  |  |  |  |  |
| Condyle hypoplasia |  |  |  |  |  |  |  |  | X |  |  |  |  |  |  |  |  |  |  |  |  | X |  |  |  |  |  |
| Deeply arched palate |  |  |  |  |  |  |  |  |  |  |  |  | X |  |  | X |  |  |  |  |  | X |  |  |  |  | X |
| Atrophy of alveolar processes |  |  |  | X |  |  |  |  |  |  | X |  |  |  |  |  |  |  |  |  |  | X |  |  |  |  | X |
| **Orodental anomalies** |  |  |  |  |  |  |  |  |  |  |  |  |  |  |  |  |  |  |  |  |  |  |  |  |  |  |  |
| *Number* |  |  |  |  |  |  |  |  |  |  |  |  |  |  |  |  |  |  |  |  |  |  |  |  |  |  |  |
| Agenesis |  |  |  |  |  |  | X | X |  | X | X |  | X |  | X |  |  |  |  |  |  | X |  |  |  |  | X |
| *Shape* |  |  |  |  |  |  |  |  |  |  |  |  |  |  |  |  |  |  |  |  |  |  |  |  |  |  |  |
| Shovel incisors |  |  |  |  |  |  |  |  |  |  |  |  |  |  |  |  |  |  |  |  |  |  |  |  |  |  | X |
| Screw driver incisors |  |  |  |  |  |  |  |  |  |  |  |  |  |  |  |  |  |  |  |  |  |  |  |  |  |  | X |
| *Size* |  |  |  |  |  |  |  |  |  |  |  |  |  |  |  |  |  |  |  |  |  |  |  |  |  |  |  |
| Macrodontia |  |  |  |  |  |  |  |  |  |  |  | X |  |  |  |  |  |  |  |  |  | X |  |  |  |  | X |
| Microdontia |  |  |  |  |  |  |  |  |  |  |  |  |  |  |  | X |  |  |  |  |  |  |  |  |  |  | X |
| Taurodontism |  |  |  |  |  |  |  |  |  |  |  |  |  |  |  |  |  |  |  |  |  |  |  |  |  |  | X |
| Radiculomegaly |  |  |  |  |  |  |  |  |  |  |  |  |  |  |  |  |  |  |  |  |  |  |  |  |  |  | X |
| Short roots in PT |  |  |  |  |  |  |  |  |  |  |  |  | X |  |  |  |  |  |  |  |  | X |  |  |  |  |  |
| *Structure* |  |  |  |  |  |  |  |  |  |  |  |  |  |  |  |  |  |  |  |  |  |  |  |  |  |  |  |
| Enamel defects |  |  |  |  |  |  |  |  |  |  |  |  |  |  | X | X | X |  |  |  |  | X |  |  |  |  | X |
| Pulpar calcification |  |  |  |  |  |  |  |  |  |  |  |  |  |  |  |  |  |  |  |  |  |  |  |  |  |  | X |
| *Eruption* |  |  |  |  |  |  |  |  |  |  |  |  |  |  |  |  |  |  |  |  |  |  |  |  |  |  |  |
| Delayed eruption PT |  |  |  |  |  |  |  |  |  |  |  |  |  |  |  |  |  |  |  |  |  |  |  |  |  |  |  |
| Delayed eruption pt |  |  | X |  |  |  |  |  |  |  |  |  |  |  | X |  |  |  | X |  |  | X |  |  |  |  | X |
| Early eruption in pt and PT |  |  |  |  |  |  |  |  |  |  |  |  |  |  |  |  |  |  |  |  |  |  |  |  |  |  | X |
| Teeth rotation |  |  |  |  |  |  |  |  |  |  |  |  |  |  |  |  |  |  |  |  |  | X |  |  |  |  |  |
| Ectopic eruption |  |  |  |  |  |  |  |  |  |  |  |  | X |  |  |  |  |  |  |  |  |  |  |  |  |  | X |
| **Crowding** |  |  |  |  |  |  |  |  |  |  |  | X |  |  |  |  |  |  |  |  | X |  |  |  |  |  | X |
| **Dental caries** | X | X | X |  | X | X |  | X |  |  |  | X | X |  | X | X |  | X | X | X |  | X | X | X | X | X | X |
| **Periodontium** |  |  |  |  |  |  |  |  |  |  |  |  |  |  |  |  |  |  |  |  |  |  |  |  |  |  |  |
| Marginal gingivitis |  |  |  |  |  |  |  |  |  |  |  |  | X |  |  |  |  |  |  |  |  | X |  |  |  |  | X |
| **Functional defects** |  |  |  |  |  |  |  |  |  |  |  |  |  |  |  |  |  |  |  |  |  |  |  |  |  |  |  |
| Restricted mandibular range of motion |  |  |  |  |  |  |  |  |  |  |  |  |  | X |  |  |  |  |  |  |  |  |  |  |  |  |  |
| Deglutition |  |  |  |  |  |  |  |  |  |  |  |  |  |  |  |  |  |  |  |  |  |  |  |  |  |  | X |
| Mixed breathing |  |  |  |  |  |  |  |  |  |  |  |  |  |  |  |  |  |  |  |  |  |  |  |  |  |  | X |
| **Parafunction** |  |  |  |  |  |  |  |  |  |  |  |  |  |  |  |  |  |  |  |  |  |  |  |  |  |  |  |
| Bruxism |  |  |  |  |  |  |  |  |  |  |  |  |  |  |  |  |  |  |  |  |  |  |  |  |  |  | X |
| Malocclusion |  |  |  |  |  |  |  |  |  |  |  |  | X |  | X |  |  |  |  |  |  | X |  |  |  |  | X |
| Hypertrophied alveolar ridges |  |  |  |  |  |  |  |  |  |  |  |  |  |  |  |  |  |  |  |  | X |  |  |  |  |  |  |
| **Others** |  |  |  |  |  |  |  |  |  |  |  |  |  |  |  |  |  |  |  |  |  |  |  |  |  |  |  |
| Tooth loss |  |  |  |  |  |  |  |  |  |  |  |  |  |  |  |  |  |  |  |  |  |  |  |  |  | X |  |

**Additional file 5 Literature review of craniofacial and oro-dental findings in CS**

.
